# Midkine as a factor to counteract the deposition of amyloid β-peptide plaques: *in vitro *analysis and examination in knockout mice

**DOI:** 10.1186/1755-7682-4-1

**Published:** 2011-01-12

**Authors:** Hisako Muramatsu1, Katsunori Yokoi, Lan Chen, Keiko Ichihara-Tanaka, Terutoshi Kimura, Takashi Muramatsu

**Affiliations:** 1 Department of Biochemistry, Nagoya University Graduate School of Medicine, 65 Tsurumai-cho, Showa-ku, Nagoya, Aichi 466-8550, Japan; 2 Division of Disease Models, Center for Neurological Disease and Cancer, Nagoya University Graduate School of Medicine, 65 Tsurumai-cho, Showa-ku, Nagoya, Aichi 466-8550, Japan; 3 Department of Health and Nutrition, Faculty of Psychological and Physical Science, Aichi Gakuin University, 12 Araike, Iwasaki-cho, Nisshin, Aichi 470-0195, Japan; 4 Department of Health Science, Faculty of Psychological and Physical Science, Aichi Gakuin University, 12 Araike, Iwasaki-cho, Nisshin, Aichi 470-0195, Japan; 5 Peptide Institute Inc, 4-1-2 Ina, Minoh, Osaka 562-0015, Japan

## Abstract

**Background:**

Midkine is a heparin-binding cytokine involved in cell survival and various inflammatory processes. Midkine accumulates in senile plaques of patients with Alzheimer's disease, while it counteracts the cytotoxic effects of amyloid β-peptide and inhibits its oligomerization. The present study was conducted to understand the role of midkine upon plaque formation of amyloid β-peptide.

**Methods:**

A surface plasmon assay was performed to determine the affinity of midkine for amyloid β-peptide. The deposition of amyloid β-peptide was compared in the brain of wild-type and midkine-deficient mice. An effect of midkine to microglias was examined by cell migration assay.

**Results:**

Midkine bound to amyloid β-peptide with the affinity of 160 nM. The C-terminal half bound to the peptide more strongly than the N-terminal half, and heparin inhibited midkine from binding to the peptide. Pleiotrophin, which has about 50% sequence identity with midkine also bound to amyloid β-peptide. The deposition of amyloid β-peptide plaques in the cortex and hippocampus was more intense in 15-month-old midkine-deficient mice, compared to the corresponding wild-type mice. Midkine promoted migration of microglias in culture.

**Conclusions:**

These results are consistent with the view that midkine attenuates the deposition of amyloid β-peptide plaques, and thus progression of Alzheimer's disease, by direct binding and also by promoting migration of microglias.

## Background

An accumulation of amyloid β-peptide (Aβ) in plaques in brain tissue has been proposed to be the primary cause of the neurodegeneration in patients with Alzheimer's disease [1,2]. This view is supported by findings that anti-Aβ antibodies prevent or reverse the disease in mouse models of Alzheimer's disease [1,3,4], although clinical trials of anti- Aβ antibodies did not give expected results [5,6]. Therefore, factors affecting the accumulation of Aβ plaques in brain tissue are important to the treatment and prevention of Alzheimer's disease. This paper primarily examines the role of midkine, a heparin-binding cytokine [7-9], in the deposition of Aβ plaques in the mouse brain.

Midkine promotes neurite outgrowth [10], the survival of various cells including neurons [11-13] and the migration of inflammatory leukocytes [14,15] and neurons [16]. Midkine is involved in pathogenesis of malignant tumors [17] and diseases with immunological backgrounds [14,15,18-20] as well as in repair of damaged tissue [13,21].

Midkine was found to accumulate in senile plaques in the hippocampus of patients with Alzheimer's disease [22]. All patients examined so far exhibited the phenomenon. Reflecting this accumulation, serum levels of midkine were increased in about half of patients with Alzheimer's disease [23]. Midkine accumulation appears to be present even at the early stage of the disease, since elevation of serum midkine levels was observed in patients with incipient Alzheimer's disease, who exhibited normal range of the Mini-Mental State Examination scores at the time of diagnosis and serum collection, and later the decreased scores [23].

The accumulation of midkine in senile plaques does not necessary mean an involvement in the etiology of Alzheimer's disease. Indeed, Yu et al. [24] and Monji et al. [25] found that midkine inhibited amyloid β-fibril formation and Aβ -induced cytotoxicity in PC12 cells. These results indicate that midkine might counteract the actions of Aβ. Thus, we performed further studies to understand the role of midkine during the development of Alzheimer's disease. Firstly we examined binding properties of midkine to Aβ, and also studied the binding of pleiotrophin to Aβ. Pleiotrophin, alternatively called HB-GAM has about 50% sequence identity with midkine and share many of the activities with midkine [26-28]. Pleiotrophin is also deposited in senile plaques of patients with Alzheimer's disease [29]. Secondly, we used mice deficient in the midkine gene to analyze the *in vivo *role of midkine on accumulation of Aβ. Thirdly we investigated novel midkine activity, which might influence plaque accumulation of Aβ. All results indicated that midkine counteracted the deposition of Aβ plaques by direct binding and promotion of migration of microglias.

## Methods

### Chemicals

Midkine and amyloid β-peptide (human, 1-40) were purchased from Peptide Institute, Osaka, Japan. Pleiotrophin and half molecules of midkine and pleiotrophin were chemically synthesized as described before [30,31]. Mutant midkine proteins were produced by baculovirus as described before [32].

### Animals

Mice deficient in the midkine gene (*Mdk*)(MKKO) or pleiotrophin gene (*Ptn*)(PTNKO) were generated as described previously and maintained as heterozygotes by crossing with C57BL/6 mice [33,34]. Mice deficient in both *Mdk *and *Ptn *(DKO) were produced by crossing *Mdk*+/- *Ptn*+/- mice. Transgenic mice with APP expression under B6SJL background (APP transgenic mice) were purchased from Taconic, Germantown, NY. The transgenic mice with *Mdk*-/- genotype were obtained by crosses of the transgenic mice with *Mdk*+/- genotype, and crosses of the progenies. All mice (2 or 15 months old) used in the present experiments were bred and maintained with sterilized food, water, and bedding at the Animal Facility of the Nagoya University Graduate School of Medicine. Animal experiments were approved by the Committee on Animal Experiments of Nagoya University Graduate School of Medicine under the guidance set by the Ministry of Education, Culture, Sports, Science and Technology of Japan, and in accordance with the National Institute of Health Guide for the Care and Use of Laboratory Animals (NIH Publications No. 80-23) revised 1996. All efforts were made to minimize the number of animals and their suffering.

### Binding Assay of midkine to Aβ in the Biacore System

The binding of midkine to Aβ was evaluated in the Biacore 3000 System. A CM5 sensor chip coated with carboxymethyldextran was activated by injecting a solution (1:1) of 200 mM N-ethyl-N'-(3-dimethylaminopropyl) carbodiimide hydrochloride and 100 mM N-hydroxysuccinimide at a flow rate of 10 μl/min. A 70 μl aliquot of Aβ solution (50 μg/ml in 10 mM acetate buffer, pH 5.0) was injected for immobilization of Aβ. The remaining activated N-hydroxysuccinimide ester groups were blocked by injecting 1M ethanolamine hydrochloride/NaOH, pH 8.5, and washed with 10 μl of 1 M NaCl. Human midkine (hMK)(0.62 - 20 μg/ml), hMK C-half, hMK N-half, human pleiotrophin (hPTN), hPTN C-half, hPTN N-half and mouse midkine mutants (5 μg/ml) in the running buffer (10 mM HEPES-NaOH, pH 7.4, containing 0.15 M NaCl and 0.0005% Tween 20) were injected into the flow cell, and the change in resonance units was recorded. In the inhibition assay with heparin, hMK (5 μg/ml) and heparin (0 - 25 μg/ml) in the running buffer were injected into flow cell. Binding assays were performed at 25°C with a constant flow rate of 10 μl/ml in both association and dissociation phases.

### Immunohistochemistry and the counting of Aβ deposits

The old female mice (15 months) were perfused with 4% paraformaldehyde and the brains were excised. Tissues were fixed in 4% paraformaldehyde, dehydrated and embedded in paraffin. Sections were cut at a thickness of 5 μm and serial sections were mounted on slides, dried overnight and stored in an airtight box. Sections were stained with hematoxylin and eosin. For Aβ staining, sections were removed up from the serial slides and treated with 0.3% H_2_O_2 _in methanol for 30 min at room temperature, followed by 70% formic acid for 8 min at room temperature. Being washed with water, and with Dulbecco's phosphate-buffered saline (PBS) 3 times, the sections were blocked with 5% normal goat serum for 30 min and treated with a mouse monoclonal anti-Aβ antibody (6E10 or 4G8, Signet laboratories, Dedham, MA) for 2 h at room temperature, followed by biotin-conjugated anti-mouse IgG (IBL, Takasaki, Japan) and Vectastain ABC standard kit (PK-6100, Vector laboratories, Burlingame, CA). The staining was visualized with a diaminobenzidine tetrahydrochloride kit (Dako, Kyoto, Japan), and the sections were lightly counterstained with hematoxylin, dehydrated and then examined under a light microscope.

Deposits of Aβ with a width of more than 25 μm in the cerebral cortex and hippocampus were measured and counted at magnification of × 400 using a system microscope (BX41,Olympus), a digital camera (DP70, Olympus) and MetaMorph Basic (Molecular Devices). The number of sections was more than 15 per brain and the average number of Aβ plaques was calculated. Six mice per a genotype were analyzed, except otherwise specified.

### RT-PCR

The oligonucleotide primers, forward and reverse, used for amplification were as follows; Amyloid β precursor protein (APP) 5'-CTGACCACTCGACCAGGTTCTGGG-3' and 5'-CCGTTCTGCTGCATCTTGGAGAGA-3', Apolipoprotein E (ApoE) 5'-CTGACAGGATGCCTAGCCG-3' and 5'-ACTGGACCCAGCTGTTCCT-3', presenilin 1 (Psen1) 5'-GGTGGCTGTTTTATGTCCCAA-3' and 5'-ACTGAAGCCACCATCATCGTT-3', nicastrin (Nct) 5'-ACTCATCAGATTGGCTGCCAG-3' and 5'-TTGCCCAGTTCATTCCACAGT-3', β-secretase 1 (Bace1) 5'-CAGTGGGACCACCAACCTTC-3' and 5'-TCCACCGGCCGTAGGTATTG-3', neprilysin (Mme) 5'-GCAGCCTCAGCCGAAACTAC-3' and 5'-TCAGCATCCATCCAAGTAAG-3', insulin degrading enzyme (Ide) 5'-GAAGACAAACGGGAATACCGTG-3' and 5'-TGTGTTCTCCACTGGTGAATGC-3', and β-actin 5'-GTGGGCCGCTCTAGGCACCA-3' and 5'-CGGTTGGCCTTAGGGTTCAGGGGG-3'.

Total RNA from the brain was prepared as descried [33]. cDNA was synthesized from 1 μg of the RNA using a SuperScript First-Strand™ Synthesis System for RT-PCR (Invitrogen, Carlsbad,CA). After preincubation at 94°C for 5 min, PCR was performed for 25 cycles (β-actin), 30 cycles (APP, ApoE, Ide), or 35 cycles (Mme, Bace-1, Psn-1) of 30 sec at 94°C, 40 sec at 60°C, and 90 sec at 72°C, and also performed for 35 cycles of 30 sec at 94°C, 40 sec at 64°C, and 90 sec at 72°C for Nct.

### Culture of microglia and migration assay

Mouse primary microglias were isolated from primary mixed glial cell cultures, which were obtained from newborn wild-type C57BL/6 (WT) or MKKO mouse brains using the shaking-off method as described previously [35].

The migration of microglias was assayed using Chemotaxicell (Kurabo, Osaka, Japan) with pores 5 μm in diameter [14]. The lower surface of the filter was coated with 10 μg/ml of MK or poly-D-lysine in PBS. An aliquot of 1.0 × 10^5 ^microglia cells in RPMI1640 medium containing 0.3% BSA (100 μl) was placed in the upper chamber. Then, 600 μl of RPMI1640 medium containing 0.3% BSA was added to the lower chamber and incubated at 37°C for 4 h.

### Statistical analysis

Data are presented as the mean ± SEM. Statistical comparisons were performed using Student's *t*-test. P values less than 0.05 were considered significant.

## Results

### Binding of midkine to Aβ

**Figure 1:**
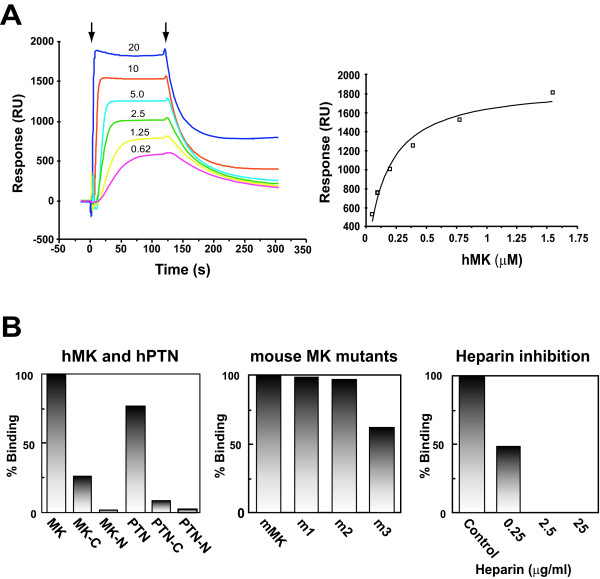
**Midkine binding to Aβ**. Specific interactions of midkine with Aβ were examined using the Biacore system. (A), Sensorgrams for the binding of human midkine (hMK) to Aβ (left) and the calibration curve (right). Various concentrations of hMK (0.62 μg-20 μg/ml) were injected onto an Aβ- immobilized sensor chip. The arrows indicate the beginning of the association phase initiated by the injection of hMK and the beginning of the dissociation phase initiated with the running buffer. RU, response units (B), Binding of hMK, hPTN (left) and mouse midkine (mMK) (middle) to Aβ, and inhibition of the binding of midkine to Aβ by heparin (right). hMK, hMKC-half (MK-C), hMK N-half(MK-N), hPTN, hPTN C-half (PTN-C), hPTN N-half (PTN-N), mMK, mMK mutants (m1: R78Q; m2: K83Q/K84Q; m3: R78Q/K83Q/K84Q) were injected onto an Aβ-immobilized sensor chip in concentration of 5 μg/ml. In the inhibition assay with heparin, hMK (5 μg/ml) and heparin (0-25 μg/ml) in the running buffer were injected into the flow cell.

Midkine inhibits the formation of amyloid β-fibril and Aβ-induced cytotoxicity in PC12 cells, and forms a complex with Aβ [24,25]. However, its binding to Aβ had not been examined in detail. We studied this point using the Biacore assay (Figure 1A). Midkine was found to interact with Aβ with a dissociation constant of 160 nM (Figure 1A). Then, we compared the binding activity at a concentration of 250 nM. Since midkine is composed of two domains, the N-terminal half and C-terminal half [7], we examined which portion bound to Aβ, and found that the C-terminal half bound more strongly (Figure 1B). The C-terminal half has two heparin-binding sites [36]. The mutation of key amino acids in either of the two sites [32] did not affect the binding activity, while the mutation of both sites resulted in a decrease in the binding activity (Figure 1B), indicating that amino acid residues in or around these heparin binding sites are involved in the binding to Aβ. This inference was consistent with the inhibitory activity of heparin toward the binding between midkine and Aβ (Figure 1B). Pleiotrophin also bound to Aβ with somewhat less activity, and its C-terminal half exhibited much less binding activity than midkine (Figure 1 B)

### Enhanced plaque deposition of Aβ in the brain of MKKO mice

**Figure 2:**
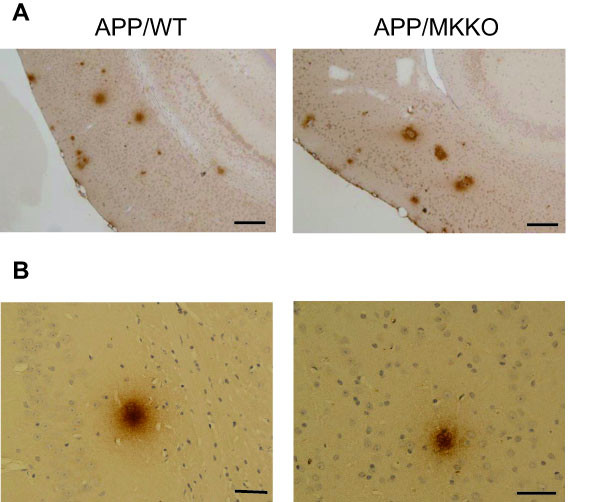
**Immunohistochemical staining of Aβ in the mouse brain.** (A) Tissue sections of the brain from APP transgenic mice (15-month-old) were stained with mouse monoclonal anti-Aβ antibody. The mice were either without midkine gene (MKKO) or with it (WT). Bar: 200 μm (B) Two examples of immunostained Aβ deposits found in the brain of 15-month-old MKKO mice without transgene. Bar: 50 μm

Deposition of Aβ plaques was observed in the cortex and hippocampus of 15-month-old wild-type mice (WT) with APP transgene (Figure 2A). We found that in the brain of the transgenic mice without MK gene (MKKO), Aβ plaques were more intense: (Figure 2A). Stronger staining and larger deposits were observed in two MKKO. Even the total number of Aβ deposit larger than 25 μm tended to be more in the MKKO. The number was 36 and 22 in MKKO, and 22 and 19 in WT.

During the study, we incidentally observed that deposits of Aβ plaques similar to those observed in Aβ transgenic WT were present in 15-month-old MKKO without transgene, although the number of the deposits were much less (Figure 2B). These deposits were never found in younger mice after repeated examination, but were detected even in 15 month-old WT with further less intensity and number. We used 6E10 antibody, which preferentially reacts with human Aβ, and cross-reacts with mouse Aβ at high antibody concentration. 4G8 antibody, which reacts mouse Aβ more intensely, revealed deposits also (data not shown). Thus, we concluded that these deposits are Aβ deposits produced with much less frequency, and performed further analysis.

**Figure 3:**
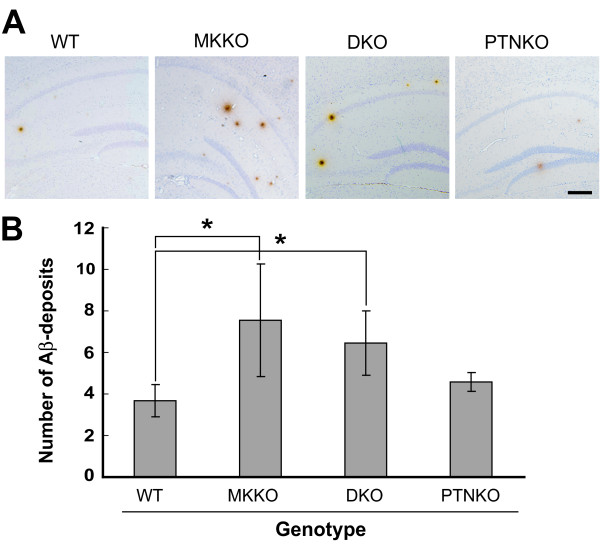
**Deposition of Aβ is enhanced in the brain of mice deficient in the midkine gene.** (A), Representative tissue section (cortex and hippocampus region) from a 15-month-old mouse brain immunostained with mouse monoclonal anti-Aβ antibody. MKKO brain sections showed an increase in immunoreactitivity for Aβ compared with WT, PTNKO or DKO brain. Bar: 200 μm. (B), Quantification of the number of Aβ plaques in A. Deposits of Aβ with a width of more than 25 μm in the cerebral cortex and hippocampus were measured and counted using a system microscope. The number of sections was more than 15 in each brain. The number of 15-months-old female mice used was 6 for each genotype, and data are presented as the mean ± S.E. *P < 0.01 compared with WT.

The total number of Aβ deposit larger than 25 μm was significantly more in MKKO compared to WT (Figure 3A, B); the number was 3.67 ± 0.87 for WT and 7.55 ± 2.90 for MKKO. However, the effect of pleiotrophin deficiency was less compared to that of midkine deficiency, and there was no statistically significant difference between WT and PTNKO. Mice deficient in both midkine and pleiotrophin (DKO) exhibited similar numbers of Aβ plaques as MKKO mice. The above observation was consistent with that in the transgenic mice and we concluded that midkine inhibited the deposition of Aβ plaques. Apparently pleiotrophin did not inhibit significantly.

**Figure 4:**
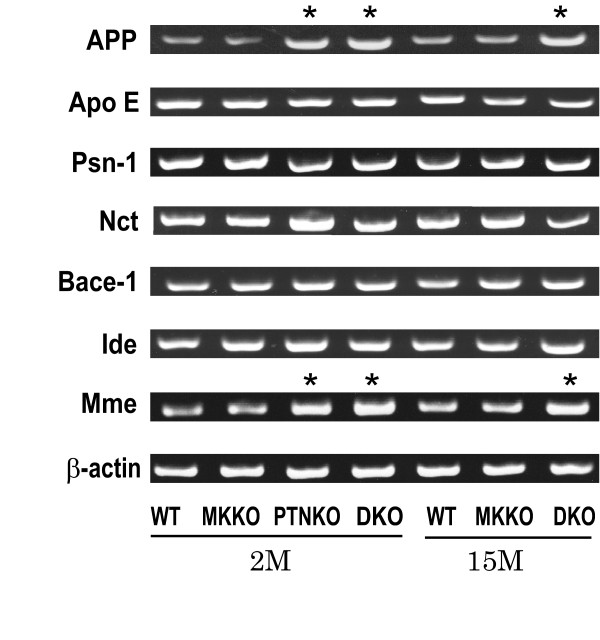
**RT-PCR analysis of the gene expression in the brain**. Aβ-related gene expression was examined using the brain of WT, MKKO, PTNKO, and DKO mice at 2 months (2M) and 15 months (15M) of age. APP: amyloid precursor protein, ApoE: apolipoprotein E, Psn-1: presenilin 1, Nct: nicastrin, Bace-1: β-secretase 1, Ide: insulin degrading enzyme (insulysin), Mme: neprilysin. *: increased expression.

We also examined the expression of genes related to etiology of Alzheimer's disease in the brain of 2- or 15-month-old mice (Figure 4). There were no significant differences in gene expression between WT and MKKO. However, increased expression of amyloid precursor protein (APP) and neprilysin (Mme) was found in the brain of PTNKO 2 month after birth, and in the brain of DKO both 2 and 15 months after birth.

### Midkine enhances the migration of microglia cells

**Figure 5:**
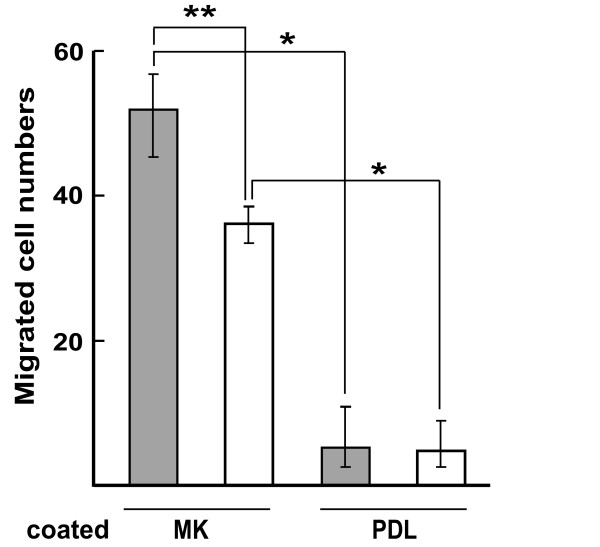
**Midkine enhances the migration of microglia cells**. The migration assay was performed with filters coated with midkine (MK) or poly-D-lysine (PDL) on the lower surface at 10 μg/ml. Microglia cells (1x10^5 ^cells in 100 μl 0.3% BSA/RPMI1640) prepared from WT (filled bars) or MKKO (open bars) newborn mice were added to the upper chamber of a Chemotaxicell, and incubated for 4 h. Ten fields (x 400 magnification) per filter were counted to determine the number of migrated cells. (1 field corresponds to 1/160 of the entire surface of the filter). The values are the mean ±S.E. per field. n = 3. *P < 0.0001, **P < 0.001.

To understand further the reason for the increased deposition of Aβ plaques in midkine-deficient mice, we investigated whether midkine enhances the migration of microglia cells. These cells in the brain mediate various inflammatory processes and are also involved in the clearance of Aβ [37]. We found that a coating with midkine enhanced the migration of microglia cells intensely compared with a coating with poly-D-lysine (Figure 5). The migratory activity of cells from MKKO was slightly less extensive than that of cells from WT.

## Discussion

Deposition of Aβ plaques in the brain of aged MKKO was more extensive compared to aged WT. This tendency was found using APP transgenic mice, and a supportive evidence was obtained by examining spontaneous deposition of a small number of Aβ plaques in 15-month-old mice without APP transgene. Therefore, midkine is likely to inhibit the process leading to the onset of Alzheimer's disease. One basis for midkine's inhibition of the deposition of Aβ plaques is its binding to Aβ; this binding can inhibit the polymerization of Aβ [24,25].

We are aware of the fact that spontaneous deposition of Aβ plaques was not reported in aged nontransgenic mice. However we should emphasize that such a plaque deposition in a small number was first detected by examining MKKO, and comparative studies in WT revealed further small number of the plaque. Probably such a small number of the plaque has been considered to be nonspecific.

Pleiotrophin, which shares much of its activity with midkine [7,28,29], was observed to bind Aβ with somewhat decreased activity compared to midkine. Even though expression of APP and Mme was increased in PTNKO, PTNKO did not exhibit a significant increase in deposition of Aβ plaques. Further, DKO did not accumulate more Aβ plaques than MKKO. The most likely explanation for this is the weaker affinity of pleiotrophin to Aβ.

We found that heparin inhibited the binding of midkine to Aβ. Since the mutation of all heparin-binding sites in midkine reduced its binding to Aβ, and the C-terminal half of midkine, which has the principal heparin-binding site [38], significantly retains the Aβ binding activity, it is likely that the binding with Aβ involves amino acids in or around the heparin-binding sites of midkine. Thus, one possible reason for the inhibition by heparin is occupation of the Aβ-binding site in midkine by heparin. In other words, heparin and Aβ may compete for a binding region on the surface of midkine. On the other hand, as heparin and other glycosaminoglycans bind to Aβ and promote its aggregation [39], another reason for the inhibition by heparin is the occupation of the midkine-binding site in Aβ. In any event, it is clear that midkine and glycosaminoglycans, which have strong affinity to each other, play opposite roles in accumulation of Aβ plaques.

That the C-terminal half of midkine is principally responsible for binding to Aβ is a key to understand the lesser affinity of pleiotrophin to Aβ compared to midkine, even though both midkine and pleiotrophin strongly bind to heparin. The different binding capability to Aβ was more evident in the C-terminal half. The C-terminal half of midkine binds to heparin strongly, but its N-terminal half only weakly [38]. However, both the N-terminal and C-terminal domains of pleiotrophin bind to heparin with similar affinities [40]. As to a structural difference of the C-terminal halves between midkine and pleiotrophin, we are aware that a heparin-binding site, Cluster 2, [32,36] is present in human and mouse midkine, but absent in pleiotrophin. The structural features of the C-terminal half of midkine, which allow strong binding to heparin, appear to cause stronger binding of midkine to Aβ compared to pleiotrophin.

The dissociation constant between midkine and Aβ was 160 nM. To evaluate the *in vivo *role of this binding, it might be necessary to estimate the concentration of midkine in the brain. Although midkine is not strongly expressed in the adult brain, it is abundant in the embryonic brain. The amount of midkine in the brain of day 12 mouse embryos was determined to be about 20 μg per g [41]. Since staining intensity of midkine in senile plaques was similar to that in the embryonic brain, we infer that a similar level of midkine is locally present in the plaques. This inference makes the dissociation constant to be significant *in vivo. *It should also be mentioned that the dissociation constant of midkine and heparin determined by the same method was 159 nM [42].

Midkine, Aβ, heparan sulfate proteoglycans with heparin-like domains and other molecules coexist in the brain tissue and senile plaques. Thus, the interactions between midkine and Aβ are affected by other molecules. When we consider interactions between midkine, Aβ and heparan sulfate proteoglycans, the relative concentration of heparan sulfate proteoglycans to Aβ is important because of similar levels of the affinities of heparin and Aβ to midkine. When the concentration of heparan sulfate proteoglycans is low, the binding of Aβ and midkine will become prominent, and aggregation inhibitory activity of midkine [25] may be exerted *in vivo*.

We also found the migration of microglias to be enhanced by midkine. Microglias are involved in the clearance of Aβ [37]. Thus, the promotion of microglial migration would be another reason why midkine suppresses accumulation of Aβ. However, some caution is required to state that enhancement of migration of microglias by midkine contributes to suppression of the disease progression, since there are reports that microglias are involved in inflammation in the brain, and in etiology of Alzheimer's disease [43,44].

## Conclusions

Midkine suppressed the accumulation of Aβ plaques as evidenced from the phenotype of MKKO. The basis of this midkine activity was relatively strong binding of midkine to Aβ. Furthermore, midkine promoted the migration of microglias. Midkine is expected to suppress the process leading to Alzheimer's disease.

## Abbreviations

Aβ, amyloid β-peptide; APP, amyloid precursor protein; DKO, double knockout; hMK, human midkine; hPTN, human pleiotrophin; MKKO, midkine knockout; mMK, mouse midkine; Nct, nicastrin; PTNKO, pleiotrophin knockout; WT, wild-type

## Competing interests

The authors declare that they have no competing interests.

## Authors' contributions

HM designed all experiments, performed immunohistochemical analysis, PCR and surface plasmon resonance and prepared significant portion of the first draft of the manuscript, KY performed immunohistochemical analysis at the earlier stage of this work, LC performed migration assay of microglias, KI participated in immunohistochemical analysis and prepared midkine mutants, TK prepared midkine half molecules and pleiotrophin, TM continued discussion throughout the course of the work, prepared a portion of the first draft and the major portion of the second draft. All authors contributed to the preparation of the manuscript and approved the final version.
